# Long-term outcomes with non-dose dense chemotherapy for Ewing sarcoma – a follow up of the cohort treated with EFT-2001 protocol

**DOI:** 10.3332/ecancer.2025.2007

**Published:** 2025-10-07

**Authors:** Badira Cheriyalinkal Parambil, Girish Chinnaswamy, Maya Prasad, Venkata Rama Mohan Gollamudi, Ajay Puri, Ashish Gulia, Sajid Qureshi, Prakash Nayak, Manish Pruthi, Siddhartha Laskar, Nehal Khanna, Jifmi Jose Manjali, Amit Janu, Sneha Shah, Nilendu Purandare, Mukta Ramadwar, Poonam Panjwani, Bharat Rekhi, Pallavi Rane, Shripad Banavali

**Affiliations:** 1 Department of Pediatric Oncology, Tata Memorial Hospital, Homi Bhabha National Institute (HBNI), Mumbai 400094, India; 2 Department of Surgical Oncology, Tata Memorial Hospital, Homi Bhabha National Institute (HBNI), Mumbai 400094, India; 3 Department of Radiation Oncology, Tata Memorial Hospital, Homi Bhabha National Institute (HBNI), Mumbai 400094, India; 4 Department of Radiodiagnosis, Tata Memorial Hospital, Homi Bhabha National Institute (HBNI), Mumbai 400094, India; 5 Department of Nuclear Medicine, Tata Memorial Hospital, Homi Bhabha National Institute (HBNI), Mumbai 400094, India; 6 Department of Pathology, Tata Memorial Hospital, Homi Bhabha National Institute (HBNI), Mumbai 400094, India; 7 Clinical Research Secretariat, Tata Memorial Hospital, Homi Bhabha National Institute (HBNI), Mumbai 400094, India

**Keywords:** non-dose dense chemotherapy, Ewing sarcoma, cardiotoxicity, long-term outcomes

## Abstract

Collaborative trials of co-operative groups have currently established interval compressed chemotherapy (ICC) as the standard of care, though there are concerns regarding the application of the same in low middle-income countries (LMICs). This study is a retrospective analysis of the long-term outcomes of a follow-up cohort (*n* = 200), constituted by patients (<15 years) with Ewing sarcoma (ES) treated with curative intent (including localised and metastatic patients) during January 2013–June 2017 on a non-dose dense chemotherapy protocol, EFT-2001. Local therapy was planned at 9–12 weeks of therapy and was delivered in all but three patients who had events before local control. At a median follow-up of 97 months (95%CI:91–103 months), 7-year event-free survival (EFS) and overall survival (OS) of the whole cohort were 55% (95%CI:49%–63%) and 69% (95%CI:63%–76%), respectively. Seven-year EFS and OS for the localised cohort were 60% (95%CI:53%–69%), 73% (95%CI:66%–80%) and for metastatic cohort were 37% (95%CI:24%–55%) and 53% (95%CI:39%–72%), (*p* = 0.003, *p* = 0.015), respectively. Non-relapse mortality was 8% (*n* = 16). Anthracycline dose, axial location, poor histological necrosis and older age group were associated with adverse outcomes. Cardiotoxicity was reported in 13%, with one-third developing symptomatic cardiac dysfunction. Long-term outcomes for children with ES treated on a non-dose dense chemotherapy protocol, in the setting of a higher treatment-related mortality, have relatively fair outcomes, though suboptimal compared to the ICC approach. ICC could be introduced in a phased manner in high-risk subsets in LMICs with better resources and an active nutritional rehabilitation and supportive care programme, while EFT-2001 protocol still could be a practical solution in resource-constrained settings.

## Introduction

Treatment of Ewing sarcoma (ES) has witnessed major collaborations of co-operative groups across continents, addressing different drug combinations and schedules over the last few decades. These trials have established interval compressed chemotherapy (ICC) vincristine, doxorubicin, cyclophosphamide/ifosfamide, etoposide (VDC/IE) as the current standard of care in ES, especially in localised tumours [1,2]. Relative to the standard timing chemotherapy (STC), applied 3 weekly, analysis of long-term outcomes showed improved survival with ICC with no significant increase in toxicities [3]. Non-dose dense chemotherapy has been an adaptive choice for low middle-income countries (LMICs), where the application of ICC is impeded by several concerns [4, 5]. Limited access to aggressive, supportive and intensive care facilities, undernutrition leading to treatment-related morbidity (causing treatment interruptions and delays) and mortalities, increased background prevalence of multi-drug resistant bacterial infections and increased incidence of certain acute and late effects of therapy (especially anthracycline-induced cardiotoxicity) are reasonable concerns in the population, restricting application of ICC. This has led to the development and continued use of non-dose dense chemotherapy protocols to optimise survival as a feasible and practical strategy in developing countries. EFT-2001, is such an indigenous chemotherapy protocol developed in our institution, the outcomes of which have been published earlier [4]. This study analyses the long-term outcomes and prognostic factors of the same cohort.

## Materials and methods

### Aims and objectives

Patients accrued over a 4½-year period, treated by a uniform non-dose dense chemotherapy protocol, were studied retrospectively for long-term outcomes and prognostic factors. Primary objectives were to assess event-free survival (EFS) and relapse-free survival (RFS). Secondary objectives were to assess overall survival (OS) and to identify prognostic factors and delineate cardiotoxicity.

### Methods

Treatment-naïve children ≤15 years of age with biopsy-proven ES treated at our institute from January 2013 to July 2017 were retrospectively analysed. Diagnosis was based on morphology and immunohistochemistry (CD99, FLI-1, NKX 2.2), with molecular analysis by EWSR1 break-apart fluorescent *in situ* hybridisation in a few selected cases only. Patients were staged by Fluoro Deoxy Glucose (FDG) Positron emission computerised tomography (PET-CT) as localised or metastatic. Baseline magnetic resonance imaging (MRI) of the primary lesion was performed for all extremity tumours and selected axial tumours, as clinically indicated. In the metastatic cohort-only lung, lymph-node and oligo-bone metastases (defined as ≤3 sites) treated with a curative intent were included in this study. All received EFT-2001 chemotherapy protocol (Supplementary Figure S1) [4]. The cumulative dose of doxorubicin was reduced from 360 mg/m^2^ (Doxo_360_ cohort) to 300 mg/m^2^ (Doxo_300_ cohort) in the interim period of the study based on an internal audit of increased cardiotoxicity with the higher dose. This was planned in January 2015 and implemented from July 2015 onwards.

A response assessment imaging was performed after four cycles of chemotherapy. This evaluation included an MRI of the primary tumour site and an FDG-PET CECT scan for cases that were metastatic at presentation. Local treatment was planned after 9–12 weeks of chemotherapy by a multi-disciplinary team, with metastatic sites addressed with radiotherapy. Radiotherapy to the primary was delivered at a dose of 55.8 Gy in 31 fractions at 1.8 Gy per fraction, 5 days a week for local treatment, if it was the only modality used (definitive RT), and at 45 Gy for post-operative radiotherapy (PORT) at 1.8 Gy per fraction. The decision for PORT to the primary was taken by the same team on a case-by-case basis. The factors considered for PORT were poor histological response (defined as <90% necrosis in post induction surgical specimen), positive or close margins, baseline pathological fracture or large soft tissue component, nerve encasement, intra-operative contamination as assessed by onco-surgeon or primary site-chest wall, head and neck, pelvis. All those with lung metastases received lung bath to a dose of 12.6 Gy for seven fractions irrespective of the response and were delivered at the time of definitive RT or following surgery to the primary. If gross residual lung metastasis were present post induction and was surgically resectable, metastatectomy was contemplated prior to lung bath. Bone metastasis was addressed with RT at a dose of 55.8 Gy to the metastatic site, and lymph node metastasis received RT to the lymph node region at a dose of 45 Gy. Details of the therapy delivered and the study population are also available from the earlier publication [4].

### Statistical methods

Baseline variables and outcomes were analysed by descriptive statistics. For survival analysis, an event was defined as progression, relapse, development of second malignancy or death due to any cause. EFS was calculated from the date of diagnosis to event or last follow-up, RFS from the date of diagnosis to relapse/progression or last follow-up and OS from the date of diagnosis to death due to any cause or last follow-up. Estimates of survival were computed using the Kaplan–Meier method. The hazard ratios (HRs) and significance associated with patient characteristics were assessed in a Cox proportional hazards regression model. In multivariate analysis, we included variables with a *p* value <0.1 on univariate analysis. A *p* value ≤0.05 was considered significant. Statistical analysis was performed using STATA software, version 16.1.

## Results

### Demographics

**Figure 1.**
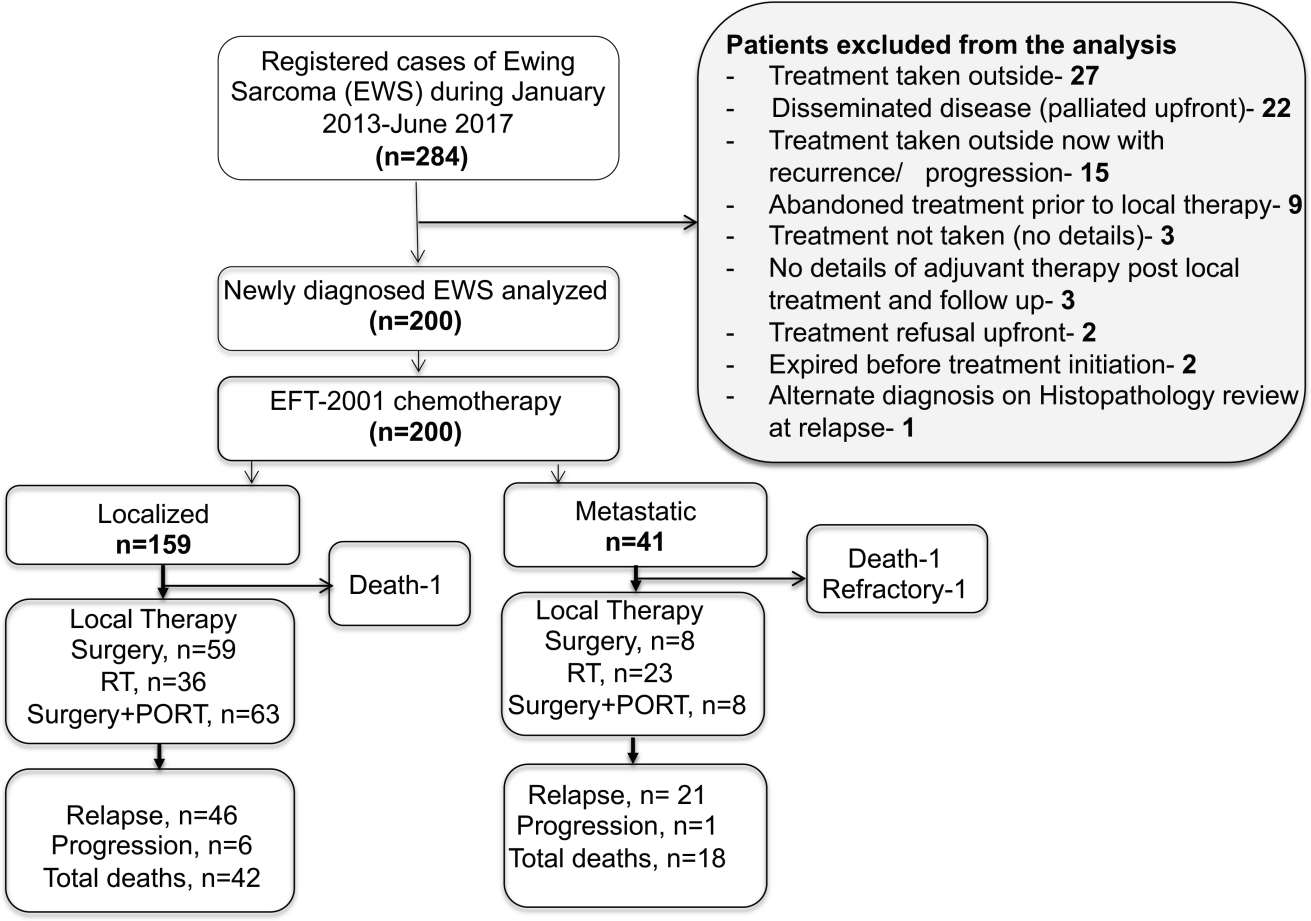
Flow diagram of this retrospective study.

Of the registered 284 children with ES during January 2013–June 2017, 200 children were eligible for the analysis. Of the 84 patients excluded, half had received treatment elsewhere: 32% sought only a second opinion with no records of chemotherapy or local therapy, and an additional 17.8% presented to us after being treated elsewhere with recurrent or progressive disease. Meanwhile, 26% of the excluded group were managed with palliative intent due to extensively disseminated disease and, therefore, were not included in this analysis. Also, 13% of these excluded patients either refused treatment upfront or abandoned treatment after initiation. Flow diagram in Figure 1. There was a significant proportion of children <10 years of age with ES (41%, *n* = 82). Metastatic disease was present in 20.5% (*n* = 41), with the lung being the major site of metastases. Details in Table 1.

### Treatment

Local therapy was delivered in all but three patients who had events before local control (localised disease-1, metastatic-2). This was surgery in 33.5% (*n* = 67), definitive radiotherapy in 29.5% (*n* = 59) and surgery and PORT in 35.5% (*n* = 71) patients. In localised cohort, the distribution was: surgery – 37.3% (*n* = 59), definitive radiotherapy – 22.8% (*n* = 36) and surgery and PORT in 39.9% (*n* = 63), and in the metastatic cohort this was: surgery – 20.5% (*n* = 8), definitive RT – 59.0% (*n* = 23) and surgery followed by PORT – 20.5% (*n* = 8). The metastatic sites were also addressed with local therapy (except one, who had an event before local control). All those with lung metastases received lung bath (12.6 Gy) irrespective of the response. The majority of the bone metastases were addressed with RT (55.8 Gy) and one underwent surgery. Lymph nodes were also addressed with RT (45 Gy), except one who had complete remission post neoadjuvant chemotherapy in this cohort. Of 123 patients who underwent surgery post induction and where histopathological response was assessed, necrosis was 100% in 51 patients (41.4%), 90%–99.9% in 37 patients (30%) and <90% in 35 patients (28.4%). Delay in any type of local control modality (as the first modality of local control-surgery or radiotherapy) from initiation of chemotherapy was >12 weeks in 82% (*n* = 121) and delay >16 weeks was noted in 48% (*n* = 70).

### Outcomes

At the time of analysis, 123 patients were alive and 17 were lost to follow-up (No event at last follow-up and not contactable over the phone for more than a year). There were 60 documented deaths (30%) in the entire cohort. Non-relapse mortality (NRM) was 8% (*n* = 16) (infections-8, cardiomyopathy-6 (acute while on chemo-4, early chronic-2), unknown cause-2). Of the 14 NRM with a known cause, 10 (71.4%) were in the Doxo_360_ cohort (cardiomyopathy-6, infections-3 and interstitial lung disease-1).

At a median follow-up of 97 months (95%CI:91–103 months), 7-year EFS and OS of the whole cohort were 55% (95%CI:49%–63%) and 69% (95%CI:63%–76%), respectively. Seven-year EFS and OS for the localised cohort were 60% (95%CI:53%–69%), 73% (95%CI:66%–80%) and for metastatic cohort were 37% (95%CI:24%–55%) and 53% (95%CI:39%–72%), (*p* = 0.003, *p* = 0.015), respectively. Seven-year EFS and OS varied as per the site of metastases (*p* = 0.027, *p* = 0.3). All patients with isolated bone metastasis (single bone-7, >1 bone-3) but one who had expired due to sepsis, had relapsed. There were 3 patients with lymph-node only metastasis, of whom one patient died due to cardiomyopathy. Tumour necrosis (TN), older age at presentation, axial primary and residual status on FDG-PET scan post definitive RT were associated with poor outcomes across whole, localised or metastatic cohorts.

**Figure 2.**
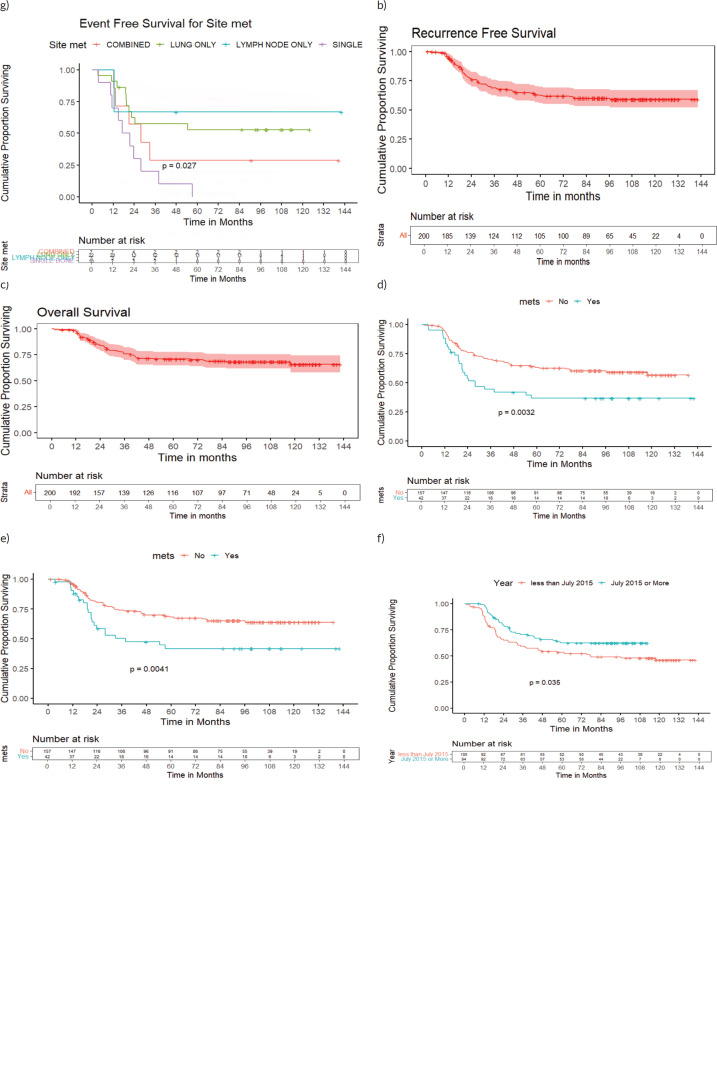
(a): EFS, (b): RFS, (c): OS of the whole cohort, (d): EFS of localised and metastatic cohort, (e): RFS of localised and metastatic cohort, (f): EFS of whole cohort-doxo_360_ and doxo_300_ subsets and (g): EFS based on the site of metastasis.

Seven-year RFS of the whole cohort, localised and metastatic cohorts were 60% (95%CI:53%–68%), 65% (95%CI:58%–73%) and 42% (95%CI:28%–61%), respectively (*p* = 0.004). Survival outcomes of various subsets are detailed in Table 2 and survival curves in Figure 2.

### Cardiotoxicity and subsequent malignant neoplasms (SMNs)

Twenty-six of 200 patients (13%) developed cardiotoxicity. Of these 26 patients, 9 (34.6%) had symptomatic cardiac dysfunction (as congestive heart failure, hypotension) and remaining 17 (65.4%) had asymptomatic cardiac dysfunction (defined as Left Ventricular Ejection Fraction <50% on 2D echocardiography) detected as part of monitoring for the same. Of the 5 deaths due to proven cardiotoxicity, 4 were in the Doxo_360_ cohort (80%). Details in Table 3.

No SMN was documented on follow-up in this cohort till the date of analysis. One patient in this cohort with sacral bone primary, who had been diagnosed as SMN in the earlier analysis, was reviewed to have the possibility of BCOR rearranged sarcoma, and hence, the event was included as relapse in the current analysis.

### Prognostic factors

Axial primary (*p* = 0.021), presence of metastasis (*p* = 0.004), presence of any residual-morphological or FDG-avid on FDG-PET CT scan done 3 months post definitive RT (*p* = 0.002, *p* ≤ 0.001) and the higher cumulative dose of doxorubicin (*p* = 0.036) were associated with poor EFS and (TN 90%–99.9%, *p* = 0.03) with improved EFS of the whole cohort on univariate analysis. Metastatic disease (*p* = 0.006) and the cumulative dose of doxorubicin (*p* = 0.037) retained significance on multivariate analysis. In the localised cohort, age at presentation (*p* = 0.019, *p* = 0.004), cumulative dose of doxorubicin (*p* = 0.02, *p* = 0.011) significantly affected EFS and RFS on multivariate analysis. Only axial primary (*p* = 0.05) was prognostic of RFS in the metastatic cohort.

Higher cumulative dose of doxorubicin (*p* = 0.012) negatively impacted OS of the whole and localised cohorts on multivariate analysis, with metastasis also (*p* = 0.011) affecting OS of the whole cohort. Details in Table 4.

## Discussion

The published data from our institute of the same cohort treated on an indigenous non-dose dense chemotherapy protocol, EFT-2001, demonstrated relatively good short-term outcomes compared to the western cohorts [4]. Three-year EFS and OS of localised ES were 70.9% and 82.8% and of metastatic disease were 42.8% and 65.3%, respectively, at a median follow-up of 42 months, with non-relapse, treatment-related mortality of 6.5% (higher than the Western world data) [2, 4, 6, 7]. The above NRM on a non-dense chemotherapy approach was a concerning issue compromising outcomes and causing apprehension about the implementation of ICC in our population. Similar NRM of 6% on a 3-weekly chemotherapy strategy has been reported from India [6]. An earlier ES cohort of 224 patients from a tertiary institute also reported a 4.5% NRM (with another 6% mortality of unknown cause) on STC [7]. This is notably higher than the dose-intense EURO-EWING99 or the dose-dense AEWS0031 Children's Oncology Group (COG) trials, which had much lower toxicity deaths of 0.6% and 0.35%, respectively [2, 8]. Similarly, only four and one deaths were documented on trial treatment in VIDE (vincristine, ifosfamide, doxorubicin, etoposide) and VDC/IE arms of EURO-EWING 2012 [1]. Subsequent to the establishment of safety and feasibility of ICC in the treatment of ES with improved efficacy in the clinical trials, ICC was adopted by certain institutes in the country, which compared the toxicities with their historical cohort treated on SCC [5]. This study, though on a smaller cohort of 125 patients, evaluated toxicities per cycle of chemotherapy delivered and observed significantly increased treatment-related toxicities, including febrile neutropenia, mucositis, transfusion support, episodes and number of hospitalisation days. Survival outcomes across both groups were comparable, albeit a shorter follow-up. Non-dose dense or STC strategy seemed appealing as a feasible and practical option in resource-constrained LMICs with challenges as elucidated before and was continued at our institution as an adaptive strategy till date, despite the results of EURO-EWING 2012 [1].

We evaluated the same cohort for long-term outcomes, including EFS, RFS, OS and major documented toxicities in this study. Seven-year EFS of 60% for the localised cohort in our study is comparable to the 10-year EFS on STC (64%), though suboptimal than the 73% EFS documented on ICC in children <18 years of age with localised disease on AEWS 0031 trial [3]. Seven-year RFS of 65% for this subset in our study elucidates the disease control better than EFS, which factored a higher NRM in our cohorts. There was a significant distinction in the outcomes of the two cohorts treated with different anthracycline doses, manifesting a better EFS and RFS in the Doxo_300_ subset. This dichotomisation was independently prognostic in both the whole and localised cohorts for EFS and RFS, partly contributed by the increased cardiotoxicity and NRM in the Doxo_360_ subset. This emphasises the importance of enhancing supportive care services and, where possible, incorporating pharmacogenomics into clinical care prior to the widespread use of ICC across all population subsets.

Tumour size was not prognostic for EFS, probably due to the application of PORT in a substantial proportion of these patients (there were broader indications of PORT in our study cohort, apart from poor TN, which was decided on a case-to-case basis in the multidisciplinary tumour board. In our localised disease cohort, 44.9% patients received PORT despite a good TN compared to 24% patients in the EURO-EWING99 R1 trial) [4, 9]. A 7-year RFS of 60% for localised tumours ≥8 cm is better than the 10-year EFS of 46% on SCC for patients with tumours ≥200 mL in the AEWS0031 COG trial, but less than the observed outcome of 74% on ICC arm, suggesting a potential high-risk subset for optimisation of outcomes [3]. Those who had 90%–99.9% TN fared best and were prognostic of EFS, though not for RFS, possibly due to the delivery of PORT in a substantial number of patients. The presence of 100% TN did not offer any survival advantage in our cohort, contrary to what is known; the reason for this is unclear [3]. Axial primary also portended suboptimal outcomes (our earlier analysis did not show any prognostic significance for the type of local therapy to the primary tumour) in the whole cohort, as did ES in children older than 10 years of age in various subsets [4].

The 7-year EFS and OS of metastatic disease at 37% and 53% have not significantly changed from the short-term 3-year EFS and OS of 43% and 65%, though differential outcomes based on the site of metastasis were notable. While those with lung-only metastasis fared relatively well, outcomes were dismal for those with isolated bone metastasis. Though limited by the sample size of (*n* = 10), all the patients but one who expired due to sepsis relapsed. This included those with even isolated single-site bone metastasis and only one patient could be salvaged till date. This is in contrast to what was observed on the analysis of 3-year outcomes in our institutional as well as western cohorts, which showed a 3-year EFS of 61% versus <20% for single-site versus >1 site of bone metastases [4, 10, 11]. There are no long-term cohorts available for comparison and though limited by sample size, this could be a factor to consider while triaging patients for treatment and resource allocation in LMICs.

A conspicuous increase in the short-term cardiotoxicity was noticed in our earlier analysis, accounting for 3% mortality in our cohort compared to none documented in the dose compressed COG trial arm to 2.5% asymptomatic left ventricular shortening with VIDE chemotherapy [2, 4, 8]. This was despite the lower cumulative doses of anthracyclines in the EFT-2001 protocol, which necessitated a dose reduction from 360 mg/m^2^ (cumulative dose) in the earlier cohorts to 300 mg/m^2^ subsequently [4]. At long-term follow-up, 13% were registered to have cardiotoxicity with nearly, one-third of them presenting with features of cardiac failure. This aligns with our published data on cardiotoxicity in a larger cohort of long-term ES survivors, which also identified undernutrition as a significant adverse factor contributing to cardiotoxicity—a concern that is particularly relevant in the context of low- and middle-income countries [12]. There is no long-term cardiotoxicity data available on the COG/EURO-EWING 2012 trial cohorts receiving a higher cumulative dose of 375 mg/m^2^ on the dose-compressed arm for comparison. This warrants investigation prior to application of ICC, which has higher doses of anthracyclines in our population, as acute proven cardiotoxicity contributed to 2.5% mortality in the whole cohort. None of the patients had SMN in our cohort on this protocol with lower cumulative doses of alkylating agents and etoposide compared to the 10-year cumulative incidence of 3.2% for patients receiving ICC versus 4.2% for patients receiving STC for all types of SMNs on EURO-EWING 2012 trial [3].

The study has its own limitations, apart from being a retrospective analysis. This is a cohort from a single referral center, that caters largely to the children from low and middle socio-economic status from across the country. A substantial majority of these children are malnourished, which plays a pivotal role in the treatment-related toxicities, despite a proactive nutritional rehabilitation programme. Moving forward, in addition to the above nutritional support, pharmacogenomic studies, optimisation of infection prevention and control strategies would go a long way in mitigating the treatment-related morbidities and mortalities. ICC could be introduced in our population with the above active interventions in a phased manner, especially in the high-risk subsets (age ≥10 years, axial tumours, metastatic disease), primarily with scrutiny for safety, tolerability, compliance and survival outcomes. Reflective of a real world LMIC setting, EFT-2001 still could be a practical solution in resource-constrained LMICs with a higher treatment-related mortality and limited supportive care facilities.

## Conclusion

Long-term outcomes for children diagnosed with ES treated on a non-dose dense chemotherapy protocol, in the setting of a higher treatment-related mortality has relatively fair outcomes, though suboptimal compared to ICC approach. ICC could be introduced in a phased manner in high-risk subsets in LMICs with better resources and an active nutritional rehabilitation and supportive care programme, while EFT-2001 protocol still could be a practical solution in resource-constrained settings.

## List of abbreviations

EFS, Event-free survival; OS, Overall survival; PET-CT, Positron emission tomography-computed tomography; PORT, Post-operative radiotherapy; RFS, Relapse-free survival; RT, Radiotherapy; SMN, Subsequent malignant neoplasm.

## Conflicts of interest

The authors declare that there are no conflicts of interest.

## Funding

Nil.

## Data availability statement

The data that support the findings of this study are available from the corresponding author upon reasonable request.

## Figures and Tables

**Table 1.** Patient demographics and clinical characteristics.

Demographic or clinical characteristic	Number of patients (%)
Age at diagnosis, years	
≤5	33 (16.5)
6–9	49 (24.5)
≥10	118 (59.0)
Sex	
Male	112 (56.0)
Female	88 (44.0)
Primary site	
Axial	86 (43.0)
Extremities	106 (53.0)
Extra-osseous (EO)	8 (4.0)
Specific primary site	
Skull	2 (1.0)
Spine	13 (6.5)
Chest wall	27 (13.5)
Scapula	4 (2.0)
Clavicle	4 (2.0)
Humerus	25 (12.5)
Radius, ulna	8 (4.0)
Pelvis	31 (15.5)
Femur	30 (15.0)
Tibia	22 (11.0)
Fibula	12 (6.0)
Mandible	4 (2.0)
Maxilla	2 (1.0)
Hand and foot	8 (4.0)
Axilla (EO)	2 (1.0)
Lung, mediastinum (EO)	2 (1.0)
Paravetebral (EO)	2 (1.0)
Pelvis (EO)	1 (0.5)
Hand (EO)	1 (0.5)
Metastases	
Yes	41 (20.5)
No	159 (79.5)
Site of metastases (*n* = 41)	
Lung only	21 (51.2)
Bone only (single bone-7, >1 bone-3)	10 (24.4)
Lymph node only (regional)	3 (7.3)
Combined	7 (17.1)
Tumor size	
≥8 cm	127 (63.5)
<8 cm	73 (36.5)
Doxo_360_ cohort	106 (53.0)
Doxo_300_ cohort	94 (47.0)
Median time to delivery of any type of local therapy (weeks)	16 (IQR:13–19)
Delay in any local control >4 months (first modality of local therapy)	
For the entire cohort (*n* = 193)	62 (32.1)
For the localised cohort (*n* = 152)	43 (28.3)
Post definitive RT 3months FDG PET scan residual (*n* = 56)	
No residual	32 (57.1)
Morphological only residual	21 (37.5)
FDG-avid residual	3 (5.4)

**Table 2.** Seven-year EFS, RFS and OS in the subsets of ES.

Subset	7-year EFS			7-year RFS			7-year OS		
	%	95% CI	*p* value	%	95% CI	*p* value	%	95% CI	*p* value
Site of metastases									
More than one site	29	8.9–92	0.027	34	11–100	0.015	36	12–100	0.3
Lung only	53	35–79		57	39–83		66	48–90	
Lymph-node only	67	30–100		100	100		67	30–100	
Bone	0	0		0	0		27	8.4–86	
TN									
Whole cohort <90%	49	35–69		66	50–86		62	48–82	
90%–90.99%	75	62–91	0.03	75	62–91	0.3	80	68–94	0.11
100%	61	49–77	0.15	63	51–78	>0.9	74	62–88	0.2
Localised cohort <90%	54	38–76		75	59–95		66	51–87	
90%–90.99%	77	64–92	0.074	77	64–92	0.8	79	67–94	0.3
100%	63	49–80	0.3	64	51–81	0.5	74	61–89	0.3
Based on doxorubicin dose									
Whole cohort Doxo_300_	49	40–60		54	45–65		78	70–87	
Doxo_360_	62	53–73	0.036	68	58–78	0.039	62	53–72	0.012
Localised cohort Doxo_300_	70	60–82		76	67–87		81	72–91	
Doxo_360_	53	43–65	0.026	56	46–69	0.015	67	57–78	0.040
Post definitive radiotherapy 3-month PET status									
Whole cohort (*n* = 56)									
No residual	65	51–84		67	53–86		84	72–98	
Morphological only residual	24	11–54	0.002	28	13–61	0.003	41	23–71	0.003
FDG-avid residual	0	0	<0.001	0	0	<0.001	0	0	<0.001
Localised cohort									
No residual	82	66–100		82	66–100		94	84–100	
Morphological only residual	26	10–64	0.002	28	11–67	0.002	48	27–85	0.021
FDG-avid residual	0	0	<0.001	0	0	<0.001	0	0	0.006
Metastatic cohort									
No residual	45	25–80		48	28–85		71	51–99	
Morphological only residual	20	3.5–100	0.3	30	6.3–100	0.4	20	3.5–100	0.048
FDG-avid residual	0	0	0.027	0	0	0.029	0	0	-

EFS- Event Free Survival, RFS- Relapse Free Survival, OS- Overall Survival, PET- Positron Emission Tomography

**Table 3.** Details of proven cardiotoxicity.

Characteristic	Asymptomatic		Symptomatic	
	Total	Death	Total	Death
Timing of presentation^#^				
Acute	11	0	6	3*
Early chronic	4	0	3	2
Late chronic	2	0	0	0

* One child apart from this three died from a clinical diagnosis of cardiac failure in emergency before work-up was possible

# Acute-onset of cardiomyopathy during the course of treatment, early chronic-onset within a year of completion of chemotherapy, late chronic- onset after a year of completion of chemotherapy

**Table 4.** Prognostic factors (A) EFS- univariate analysis, (B) RFS- univariate analysis, (C) OS- univariate analysis, (D) Multivariate analysis for EFS, RFS, OS.

**A)**									
**Variable**	**HR**	**95% CI**	***p* value**	**HR**	**95% CI**	***p* value**	**HR**	**95% CI**	***p* value**
	**Whole cohort**			**Localised cohort**			**Metastatic cohort**		
Gender	0.83	0.54–1.25	0.4	0.76	0.46–1.26	0.3	1.23	0.57–2.65	0.6
Axial site	1.64	1.08–2.49	0.021	1.38	0.84–2.27	0.2	2.1	0.88–5.04	0.1
Age ³10 years	1.45	0.93–2.25	0.1	1.91	1.10–3.30	0.021	0.7	0.32–1.51	0.4
Metastases	1.97	1.24–3.12	0.004	-	-	-	-	-	-
Doxo_300_ versus Doxo_360_ cohort	0.63	0.41–0.97	0.036	0.55	0.32–0.93	0.026	0.76	0.35–1.65	0.5
Local control delay >4 months	1.24	0.77–2.00	0.4	1.28	0.71–2.33	0.4	0.72	0.30–1.72	0.5
Tumor size ≥ 8 cm	1.4	0.90–2.18	0.14	1.55	0.91–2.62	0.11	0.74	0.32–1.72	0.5
TN 90–99.9%^#^	0.42	0.19–0.92	0.03	0.46	0.20–1.08	0.074	0.4	0.04-3.65	0.4
Site of metastasis (Bone versus combined)	1.92	0.65–5.63	0.2	-	-	-	1.92	0.65–5.63	0.2
Post definitive RT PET* Morphological residual FDG avid residual	3.47 19.4	1.59–7.55 4.62–81.5	0.002 <0.001	7.91 31	2.18–28.7 4.37–220	0.002 <0.001	1.91 24.1	0.57–6.44 1.44–401	0.3 0.027
**B)**									
**Variable**	**HR**	**95% CI**	***p* value**	**HR**	**95% CI**	***p* value**	**HR**	**95% CI**	***p* value**
	**Whole cohort**			**Localised cohort**			**Metastatic cohort**		
Gender	0.97	0.62–1.54	>0.9	0.89	0.51–1.55	0.7	1.47	0.64–3.42	0.4
Axial site	1.87	1.18–2.97	0.008	1.54	0.89–2.67	0.12	2.71	0.99–7.41	0.052
Age ≥10 years	1.85	1.12–3.07	0.017	2.54	1.33–4.86	0.005	0.85	0.36–1.99	0.7
Metastases	2.05	1.24–3.39	0.005	-	-	-	-	-	-
Doxo_300_ versus Doxo_360_ cohort	0.61	0.38–0.98	0.041	0.48	0.26–0.87	0.015	0.89	0.38–2.05	0.8
Local control delay >4 months	1.29	0.76–2.18	0.3	1.46	0.76–2.78	0.3	0.65	0.25–1.67	0.4
Tumor size ≥8 cm	1.32	0.82–2.14	0.3	1.45	0.81–2.57	0.2	0.7	0.29–1.73	0.4
TN 90–99.9%^#^	0.65	0.26–1.60	0.3	0.9	0.31–2.59	0.8	0.4	0.04–3.65	0.4
Site of metastasis (Bone versus combined)	2.19	0.67–7.13	0.2	-	-	-	-	-	-
Post definitive RT PET* Morphological residual FDG avid residual	3.45 25.6	1.51–7.84 5.76–113	0.003 <0.001	7.47 37.3	2.04–27.4 5.09–273	0.002 <0.001	1.71 23.1	0.44–6.74 1.38–386	0.4 0.029
**C)**									
**Variable**	**HR**	**95% CI**	***p* value**	**HR**	**95% CI**	***p* value**	**HR**	**95% CI**	***p* value**
	**Whole cohort**			**Localised cohort**			**Metastatic cohort**		
Gender	0.76	0.46–1.27	0.3	0.76	0.41–1.39	0.4	1.04	0.41–2.65	>0.9
Axial site	1.37	0.83–2.28	0.2	0.97	0.52–1.79	>0.9	2.48	0.81–7.55	0.11
Age ³10 years	1.43	0.83–2.44	0.2	1.85	0.95–3.62	0.071	0.81	0.32–2.05	0.7
Metastases	1.96	1.13–3.41	0.017	-	-	-	-	-	-
Doxo_300_ versus Doxo_360_ cohort	0.49	0.29–0.86	0.012	0.51	0.26–0.98	0.044	0.46	0.17–1.23	0.12
Local control delay >4 months	1.03	0.58–1.83	>0.9	0.90	0.43–1.84	0.8	0.79	0.27–2.32	0.7
Tumor size ≥8 cm	1.33	0.78–2.28	0.3	1.56	0.82–2.97	0.2	0.69	0.26–1.85	0.5
TN 90–99.9%^#^	0.49	0.20–1.19	0.11	0.58	0.22–1.51	0.3	-	-	-
Site of metastasis (Bone versus combined)	1.33	0.37–4.71	0.7	-	-	-	1.33	0.37–4.71	0.7
Post definitive RT PET* Morphological residual FDG avid residual	4.88 14.9	1.69–14.1 3.39–65.4	0.003 <0.001	11.8 31.5	1.45–96.6 2.69–368	0.021 0.006	4.2 -	1.02–17.4 -	0.048 -
**D)**									
**Variable**	**HR**	**95% CI**	***p* value**	**HR**	**95% CI**	***p* value**	**HR**	**95% CI**	***p* value**
	**EFS**								
	**Whole cohort**			**Localised cohort**			**Metastatic cohort**		
Presence of metastases	1.91	1.20–3.04	0.006	-	-	-	-	-	-
Axial primary	1.52	0.99–2.34	0.054				1.46	0.57–3.77	0.4
Doxo_300_ versus Doxo_360_ cohort	0.63	0.41–0.97	0.037	0.53	0.31–0.90	0.02	-	-	-
Age ≥10 years	-	-	-	1.93	1.11–3.35	0.019	-	-	-
Site of metastasis (Bone versus combined)	-	-	-	-	-	-	1.77	0.59–5.27	0.3
	**RFS**								
Presence of metastases	1.95	1.17–3.24	0.01	-	-	-	-	-	-
Axial primary	1.61	0.99–2.61	0.054	-	-	-	2.74	1.00–7.50	0.05
Age ≥10 years	1.67	0.99–2.80	0.053	2.58	1.35–4.94	0.004	-	-	-
Doxo_300_ versus Doxo_360_ cohort	0.58	0.36–0.94	0.027	0.46	0.25–0.83	0.011	0.84	0.36–1.95	0.7
	**OS**								
Presence of metastases	0.49	0.28–0.85	0.011	-	-	-	-	-	-
Doxo_300_ versus Doxo_360_ cohort	2.03	1.17–3.55	0.012	0.49	0.25–0.96	0.037	0.45	0.17–1.21	0.11
Age ≥10 years	-	-	-	1.86	0.95–3.65	0.071	2.53	0.83–7.70	0.1

* *n* = patients who received definitive RT,

# *n* = patients who underwent surgery

**Supplementary Figure S1.**
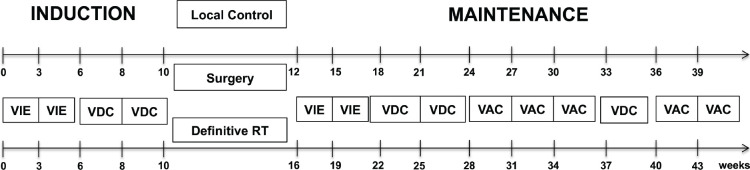
Treatment schema used in the study. EFT-2001, the chemotherapy protocol consists of 3-weekly chemo with weekly vincristine (1.5 mg/m^2^, max 2 mg D1, D8, D15 in induction and D1, D8 in maintenance cycles) except for an interval compression in induction between 2 VDC cycles. VIE: vincristine-1.5 mg/m^2^ iv push D1, ifosfamide- 2,000 mg/m^2^ iv infusion over 2 hours with iv mesna 600 mg/m^2^ at 0,3,6,9 hours of ifosfamide D1-D5, etoposide- 100 mg/m^2 ^iv over 1–2 hours D1-D5, VDC: vincristine-1.5 mg/m^2^ iv push D1, doxorubicin- 60 mg/m^2^ iv over 6 hours D1, cyclophosphamide- 600 mg/m^2^ iv over 30 minutes D1, VAC: vincristine-1.5 mg/m^2^ iv push D1, actinomycin-D- 1 mg/m^2 ^iv push D1, cyclophosphamide- 600 mg/m^2 ^iv over 30 minutes D1 . Local therapy was planned at 10–12 weeks. PORT was deivered as per indications described. Metastatic sites were addressed with radiotherapy. For those who received definitive radiotherapy, concurrent chemotherapy was given: vincristine- 1.5 mg/m^2 ^iv push D1, cyclophosphamide- 600 mg/m^2 ^iv over 30 minutes D1 ± actinomycin-D- 1 mg/m^2^ iv push D1 (actinomycin-D in concurrent chemotherapy for extremity tumours only) with weekly vincristine- 1.5 mg/m^2^ iv push on D8, D15. Radiotherapy was delivered at a dose of 55.8 Gy in 31# at 1.8 Gy/#, 5 days a week for local control if it was the only modality used (definitive RT) and at 45 Gy for PORT at 1.8 Gy/#.
